# Beyond the marrow: insights from comprehensive next-generation sequencing of extramedullary multiple myeloma tumors

**DOI:** 10.1038/s41375-024-02206-w

**Published:** 2024-03-16

**Authors:** T. Jelinek, D. Zihala, T. Sevcikova, A. Anilkumar Sithara, V. Kapustova, H. Sahinbegovic, O. Venglar, L. Muronova, L. Broskevicova, S. Nenarokov, D. Bilek, T. Popkova, H. Plonkova, J. Vrana, V. Zidlik, P. Hurnik, M. Havel, M. Hrdinka, Z. Chyra, G. Stracquadanio, M. Simicek, R. Hajek

**Affiliations:** 1 Department of Hematooncology, Faculty of Medicine, University of Ostrava, Ostrava, Czech Republic; 2 Department of Hematooncology, University Hospital Ostrava, Ostrava, Czech Republic; 3 Department of Biology and Ecology, Faculty of Science, University of Ostrava, Ostrava, Czech Republic; 4 Department of Pathology, University Hospital Ostrava, Ostrava, Czech Republic; 5 Department of Nuclear Medicine, University Hospital Ostrava, Ostrava, Czech Republic; 6 Department of Imaging Methods, Faculty of Medicine, University of Ostrava, Ostrava, Czech Republic; 7 School of Biological Sciences, The University of Edinburgh, Edinburgh, EH9 3BF UK

**Keywords:** Cancer genomics, Haematological cancer

## Abstract

Extramedullary multiple myeloma (EMM) is an aggressive form of multiple myeloma (MM). This study represents the most comprehensive next-generation sequencing analysis of EMM tumors (*N* = 14) to date, uncovering key molecular features and describing the tumor microenvironment. We observed the co-occurrence of 1q21 gain/amplification and MAPK pathway mutations in 79% of EMM samples, suggesting that these are crucial mutational events in EMM development. We also demonstrated that patients with mutated *KRAS* and 1q21 gain/amplification at the time of diagnosis have a significantly higher risk of EMM development (HR = 2.4, *p* = 0.011) using data from a large CoMMpass dataset. We identified downregulation of CXCR4 and enhanced cell proliferation, along with reduced expression of therapeutic targets (CD38, SLAMF7, GPRC5D, FCRH5), potentially explaining diminished efficacy of immunotherapy. Conversely, we identified significantly upregulated EZH2 and CD70 as potential future therapeutic options. For the first time, we report on the tumor microenvironment of EMM, revealing CD8+ T cells and NK cells as predominant immune effector cells using single-cell sequencing. Finally, this is the first longitudinal study in EMM revealing the molecular changes from the time of diagnosis to EMM relapse.

## Introduction

Extramedullary multiple myeloma (EMM) is an aggressive form of multiple myeloma (MM), the second most common blood cancer, which is characterized by the clonal proliferation of plasma cells (PCs) within bone marrow (BM) [1]. In EMM, malignant PCs become independent of the BM microenvironment and infiltrate other tissues and organs, creating soft tissue tumors [2]. Therefore, EMM is associated with treatment resistance and short survival [3, 4]. In clinical setting, it is important to distinguish EMM from the less aggressive form, paraskeletal (bone-related) MM, which manifests as the presence of soft tissue lesions adjacent to the bone [1, 5]. The incidence of EMM is reported to be 1.7–4.5% at diagnosis (primary EMM) and up to 43% at relapse (secondary EMM) [1, 6, 7]. Recent clinical trials in heavily pretreated MM patients who are “triple class exposed” (previous proteasome inhibitor [PI], immunomodulatory drug [IMiD], and anti-CD38 monoclonal antibody [mAb]) reported a much higher incidence of EMM than in the past [8, 9]. This observation is probably associated with longer survival of MM patients due to novel treatment options, as well as improved availability of modern imaging techniques, such as PET/CT. Thus, EMM is becoming a clinically relevant issue and one of the “hot topics” in the MM community alongside with circulating tumor PCs, which may be responsible for EMM spread and possess great prognostic value [10–12]. The molecular mechanisms mediating EMM development, as well as the composition of the tumor microenvironment, are still poorly understood but crucial for the efficacy of novel immunotherapy.

No detailed genomic or transcriptomic profiling has been carried out for EMM. In addition to a limited number of samples, studies focusing on chromosomal aberrations are usually biased by a preselected set of FISH targets [13, 14], and small somatic mutations are analyzed mostly with targeted panels, excluding the vast majority of human genes [15, 16]. Similarly, almost no transcriptomic data obtained directly from EMM tumor cells exist [17, 18].

In this study, we performed the most comprehensive investigation of EMM cells to date, combining data obtained by FISH, whole exome sequencing (WES), bulk RNA sequencing (RNA-seq), single-cell RNA sequencing (scRNA-seq), and flow cytometry in 14 patients with EMM. Importantly, we obtained genomic and transcriptomic data from eight paired samples of BM aberrant PCs from the time of diagnosis and compare them with EMM cells from the time of relapse which, to the best of our knowledge, represents the first longitudinal study in EMM.

## Methods

### Patients and data collection

**Fig. 1:**
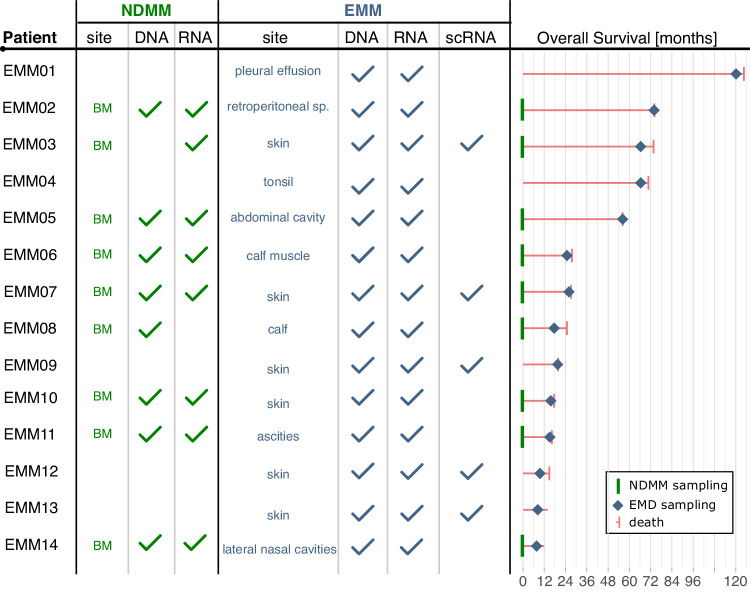
Study design. The figure illustrates the sequencing technology used for bone marrow (BM) samples from NDMM (green) and EMM (blue) patients with EMM. It also presents the overall survival with marked sampling times.

All samples were collected between 2014 and 2022 at the Department of Hematooncology, University Hospital Ostrava: 14 EMM samples from biopsies of soft tissue tumors and 14 BM samples from newly diagnosed MM (NDMM), 8 of them paired samples stored at the biobank from the time of diagnosis and 6 unpaired, and 14 unpaired BM samples from relapsed/refractory MM (RRMM) patients without evidence of EMM. All samples were used for RNA sequencing (*n* = 42). EMM and paired NDMM samples were also used for WES (*n* = 22), and some EMM samples were processed for single-cell sequencing (*n* = 5) (Fig. 1). All EMM samples were processed fresh immediately (max. 1 h) after surgery. Patients were treated in a real-world setting according to institutional guidelines. Biopsy of the soft tissue tumor was performed when clinically indicated to confirm the EMM diagnosis. The clinical characteristics of the EMM patients and treatment summaries are provided in Supplementary Table 1 and 2. The clinical characteristics of the RRMM and unpaired NDMM patients are described in Supplementary Table 3. The study was conducted in accordance with the principles of the Declaration of Helsinki and was approved by institutional ethics committee under number 511/2022. All patients provided written informed consent.

### Fluorescence in situ hybridization

For EMM tumor samples, a 2 × 5 × 5 mm slice of fresh tumor was touched several times at one spot against a methanol-cleaned uncoated slide, which was then fixed in 70% ethanol and air-dried. FISH analysis was performed using the following probes: MetaSystems XL RB1/DLEU/LAMP, XL IGH plus, XL P53, XL 1p32/1q21, XL 5p15/9q22/15q22 Hyperdiploidy, XL t(11;14), XL t(4;14), and XL t(14;16). A total of 100 cells were evaluated. FISH analysis of probe hybridization was performed with a 100× objective fluorescence microscope (Olympus BX41) with single and double emission filters. The protocol for samples from NDMM and RRMM samples is described in the Supplementary Methods.

### Fluorescence-activated cell sorting

EMM samples were collected in tubes containing normal saline and mechanically disintegrated immediately after surgery. BM aspirates from NDMM and RRMM patients were collected in tubes containing EDTA and processed within 24 h. Filtered cell suspensions from EMM and BM samples were subjected to erythrocyte lysis in NH4Cl-based lysing solution. Cells were stained with multiple fluorescence-labeled mAbs: CD38-FITC/CD45-PB/CD56-PE/CD19-PC7/CD138-APC. Cells were sorted using a BD FACSAria III (BD Biosciences) equipped with 405, 488, 561, and 633 nm excitation lasers. Pathological PCs were gated according to their immunophenotype and sorted into RPMI media containing 10% fetal bovine serum. Purity exceeded 95%.

### DNA/RNA isolation and quality assessment

Sorted aberrant PCs were subjected to DNA and RNA isolation using the AllPrep DNA/RNA Micro Kit (Qiagen, Germantown, MD) following the manufacturer’s protocol. The concentration of isolated DNA and RNA was determined on a Qubit 2.0 fluorometer (Life Technologies, ThermoFisher Scientific, Waltham, MA, USA). RNA quality was assessed on an Agilent 2200 Tapestation (Agilent Technologies, Santa Clara, CA, USA) using High Sensitivity RNA ScreenTape.

### RNA sequencing

The total RNA (5 ng) from 42 samples was used for library preparation using SMARTer Stranded Total RNA-Seq Kit v2 - Pico Input Mammalian (Takara Bio, San Jose, CA, USA). Briefly, RNA was converted to cDNA, followed by the addition of Illumina adaptors and barcodes by five cycles of PCR. Next, libraries were submitted to depletion of ribosomal cDNA using ZapR v2 enzyme and R-probes v2. Finally, libraries were amplified by 14 cycles of PCR. Libraries were pooled in equimolar ratios and the final library pool sequenced using the Illumina platform (Illumina Inc., San Diego, USA) at Macrogen Europe (the Netherlands). Sequencing resulted in an average number of 16.4 (63%), 8.0 (31%) and 1.5 (6%) million readsmapped to exonic, intronic and intergenic regions, respectively. The RNA-seq data analysis is described in detail in the Supplementary Methods.

### Whole exome sequencing

Genomic DNA from 22 samples was used for WES. For each patient, T-lymphocytes were sequenced as the normal reference. DNA (72–200 ng) from 11 patients was used for library preparation using the Twist Comprehensive (Core + RefSeq) Human Exome kit (Twist Biosciences, USA) following the manufacturer’s protocol with enzymatic fragmentation performed by Macrogen Europe (the Netherlands). Samples from three patients in the above-mentioned cohort underwent library preparation using SureSelect Human All Exon V6 (Agilent Technologies, USA), but the data were analyzed in the same way as all samples (i.e., using BED files from Twist Comprehensive [Core+RefSeq] Human Exome kit). Sequencing was performed on an Illumina platform aiming at 200× raw coverage (150 bp pair-end reads) by Macrogen Europe (the Netherlands). Sequencing resulted in an average target coverage of 94× (range 57–133×) for tumor samples and 67× (range 50–82×) for normal samples. The WES data analysis is described in detail in the Supplementary Methods.

### Single-cell analyses of EMM tumors

Single-cell RNAseq was performed using cell suspensions from five EMM tumors (5000–10,000 cells per sample) that were subjected to Chromium Next GEM Single Cell 3′ RNA reagent kit v3.1 (cat. num. 1000128, single indexing) (10x Genomics, USA) following manufacturer’s protocol. Sequencing was performed by Macrogen Europe (the Netherlands) on an Illumina sequencer. Sequencing resulted in an estimated average of 6054 cells, 34888 mean reads per cell, and 3575 median genes per cell per patient. The scRNA data analysis and flow cytometry analysis is described in detail in the Supplementary Methods.

### Survival analysis

Survival analysis of Multiple Myeloma Research Foundation CoMMpass study (NCT01454297) (“CoMMpass”, *N* = 699, IA20) is described in detail in the Supplementary methods. We used *p* = 0.05 as a threshold for significance in all analyses. We performed all computations and visualization using R(v4.0.3) and survival(v3.2.11), survminer(v0.4.9), lubridate(v1.7.10), readxl(v1.3.1), and tidyverse(v1.3.1) packages.

## Results

### Clinical characteristics of EMM relapse

**Fig. 2:**
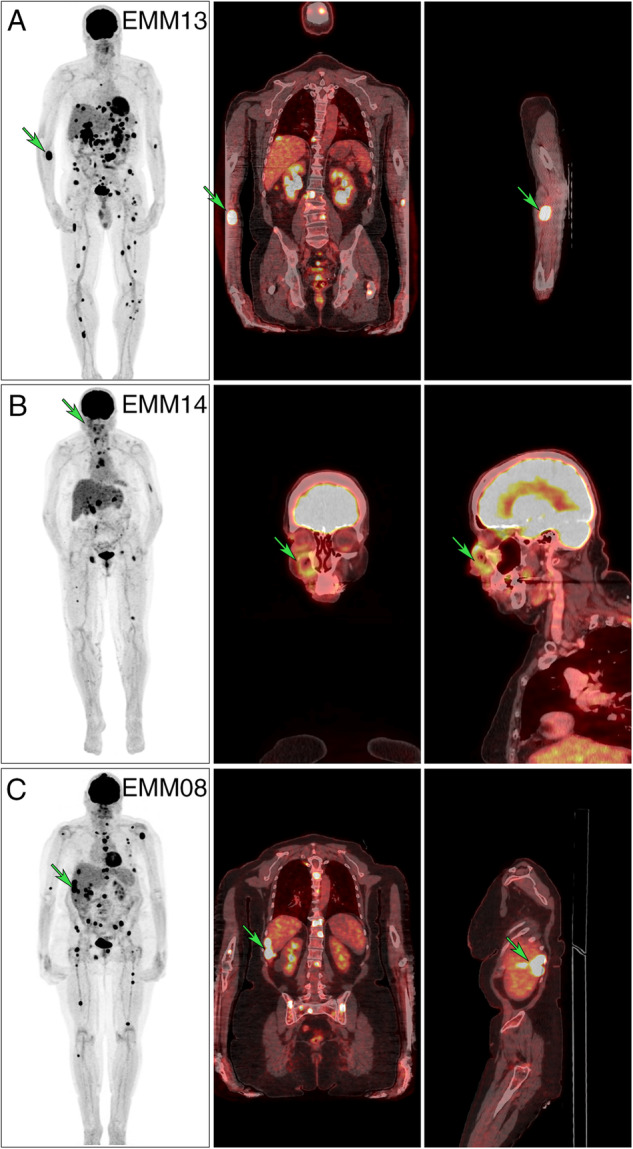
18F-FDG-PET/CT scans presenting extramedullary (EM) involvement in patients with multiple myeloma. The left column represents whole body maximum intensity projections (MIPs), the medial and right columns fused hybrid multi-planar reconstructions (MPRs) in the coronal and sagittal plane. **A** 59-year-old male with multiple metabolically active EM foci (EMM13) had a histologically evaluated lesion in the right cubital fossa (green arrow, SUVmax 17.4). **B** 75-year-old female with active foci in the skeleton and lymph nodes (EMM14) had a histologically evaluated extramedullary lesion growing near the wall of the right maxillary sinus (green arrow, SUVmax 5.1). **C** 62-year-old female with multiple active foci with liver involvement (EMM08) (green arrow, SUVmax 44.4).

A total 14 of RRMM patients (10 men and 4 women) underwent biopsy of the soft tissue tumor, which confirmed the diagnosis of EMM (Fig. 2). Median age at the time of EMM relapse was 59 years, median time from diagnosis was 22.2 months, and median overall survival from the time of EMM relapse was only 3.6 months (Fig. 1). Median number of previous lines of therapy was 3 (range 1–6), and the majority of patients received a combination of PI, IMiDs, corticosteroids, and alkylators. Six of the 14 patients had daratumumab before EMM relapse (Supplementary Table 2). Patients with EMM relapse presented with a significant drop in serum electrophoresis defined M-protein levels compared to non-EMM relapse (median 5.8 g/L vs. 14.4 g/L; *p* = 0.011) at the time of relapse, but there was no difference in free light chain levels. BM PC infiltration was significantly lower in patients with EMM relapse assessed by cytology (2.8% vs. 14.4%; *p* = 0.023) or flow cytometry (0.5% vs. 8.3%; *p* = 0.05; Supplementary Fig. 1). This minimal BM infiltration of malignant PCs translated into normal blood counts in practically all patients experiencing EMM relapse (median white blood cell count 5.1 × 10^9^ /L; median absolute neutrophil count 2.4 × 10^9^/L; median level of hemoglobin 12.0 g/dL; median platelet count 168 × 10^9^/L).

### 1q21 gain/amp is the most frequent chromosomal aberration in EMM

**Fig. 3:**
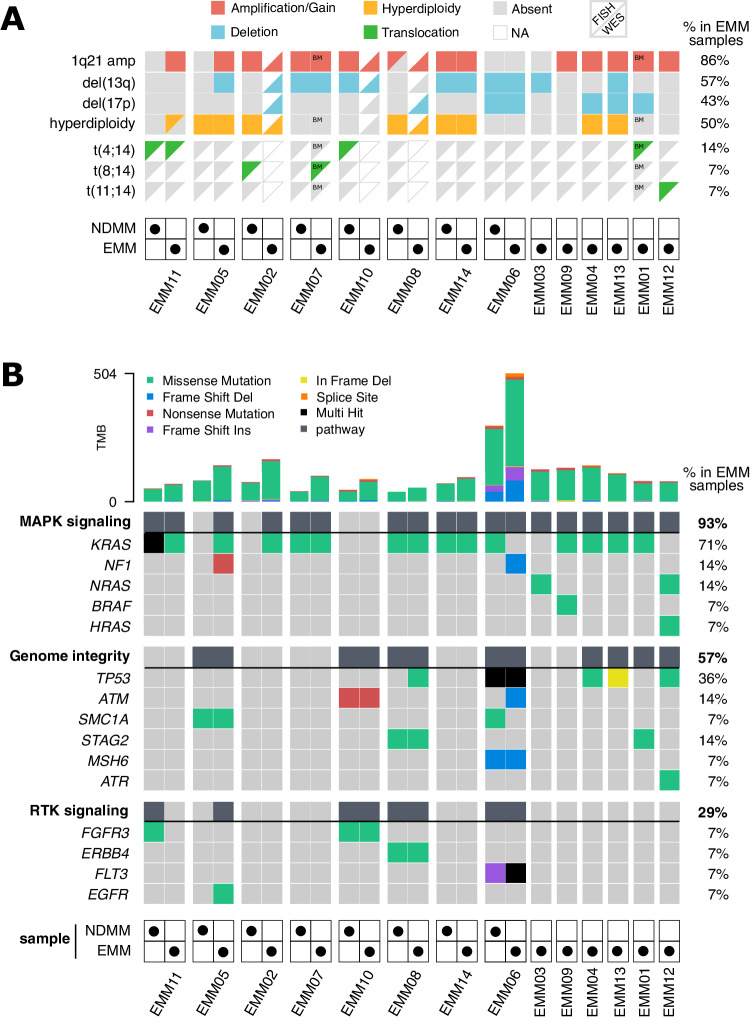
Chromosomal aberrations (CAs) and most frequently mutated pathways in EMM cells. **A** CAs are represented by two triangles: the top-left triangle depicts aberrations detected by FISH, and the bottom-right triangle denotes CAs detected by WES. The absence of a triangle indicates unavailable data. For two patients, FISH EMM samples were only available from BM and are marked as such. **B** Oncoplot produced by Maftools [61] displays the most frequently mutated pathways. Columns represent samples, and rows represent mutated genes or indicate the entire pathway (in bold text and dark grey boxes). Different types of mutations are shown in different colors. The total number of mutations is visualized at the top as a bar plot. Paired BM NDMM and EMM samples are grouped together.

Out of 14 EMM samples, we detected (by FISH and/or WES) 1q21 gain or amplification (≥4 copies) spanning the *CKS1B* gene in 12/14 samples (86%; 5 gains and 7 amplifications) (Fig. 3A). Del(13q) including *RB1* and del(17p) including *TP53* were detected in 8 (57%) and 6 (43%) cases, respectively. Surprisingly, high-risk t(4;14) was present in only 2 patients (14%). We verified this finding with RNA-seq data revealing elevated FGFR3 expression and/or presence of FGFR3—IGH fusion transcripts. Detailed copy number analysis utilizing WES data further uncovered amplifications of several oncogenes, deletions of tumor suppressor genes (TSGs) [19] and deletion of *CD38* in 4 out of 14 EMM samples, (Supplementary Fig. 2).

### Members of MAPK pathway are mutated in virtually all EMM samples

Next, we identified at least one mutated gene in the MAPK signaling pathway in almost all EMM samples (13/14, 93%; Fig. 3B), with *KRAS* as the most frequently mutated gene in our cohort (10/14, 71%; Fig. 3B, Supplementary Fig. 3). Mutations in *NF1* and *NRAS* were identified in 2 (14%) samples, and *BRAF* and *HRAS* were mutated in 1 (7%) sample. Mutations in the MAPK pathway were always clonal with the cancer cell fraction bearing them >0.8 (Supplementary Table 4), suggesting their emergence prior to extramedullary tumor formation. We also discovered that 57% of EMM samples had mutations in genes associated with genome integrity, with *TP53* being the most frequently mutated TSG (5/14; 36%). The last group of genes frequently mutated in EMM were those belonging to the receptor tyrosine kinase (RTK) signaling pathway upstream of the MAPK pathway [20]. Further, we used RNA-seq data to demonstrate that at least 80% of the above-mentioned mutations were transcribed into mRNA and are presumably translated into final protein products (Supplementary Table 4). Notably, all EMM samples with mutated *TP53* transcribed the mutated allele, with variant allele frequency (VAF) ≥ 0.9 as a sign of biallelic inactivation (Supplementary Fig. 2). All mutations in *K/N/HRAS* had VAF of at least 0.3 in the RNA-seq data. Of note, melphalan signature was detected only in 3/9 (33%) of patients that underwent ASCT (Supplementary Fig. 4).

### Combination of 1q21 gain/amp and mutated *KRAS* in NDMM patients predicts higher risk of EMM development

Due to the high frequency of 1q21 gain/amp and *KRAS* mutations in EMM, we hypothesized that NDMM patients carrying the combination of these two abnormalities are at higher risk of EMM development. We observed this combination already at the time of MM diagnosis in 3/8 (38%) paired NDMM samples (Fig. 3). Therefore, we used data collected in the CoMMpass study (*N* = 699; MM patients with all required data available) that supported our hypothesis as only the combination of 1q21 gain/amp with *KRAS* mutations (7.6% of patients), but not any of them alone (29.8% and 17.7% with only 1q21 gain/amp or KRAS respectively), resulted in a significantly higher risk of soft tissue plasmacytoma development in univariate analysis (HR = 2.7; *p* = 0.002) and multivariate Cox analysis (HR = 2.4; *p* = 0.011) with ISS, LDH level, del(13q), and del(17p) (Supplementary Table 5). In addition, we observed virtually identical results using the Fine-Gray model for survival analysis with competing risks (soft tissue plasmacytoma development vs. death, HR = 2.4; p = 0.011). No interaction was detected between *KRAS* mutations or 1q21 gain/amp with del(13q) or del(17p).

### Transcriptomic profiling of EMM cells suggests higher proliferation and decreased homing to BM

**Fig. 4:**
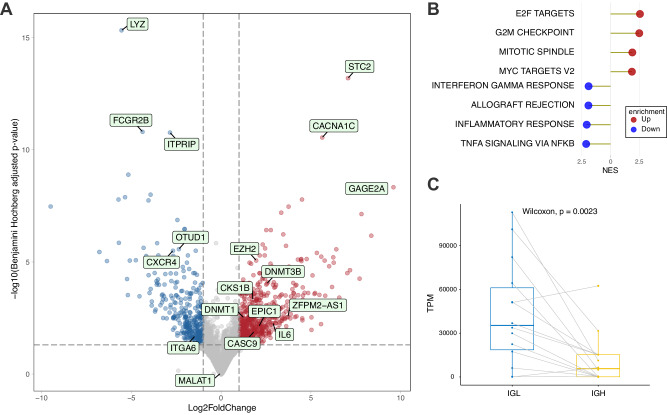
Transcriptomic profile of EMM cells. **A** Volcano plot showing the top three down- and upregulated genes, along with several other significantly deregulated genes between EMM (*N* = 14) and NDMM (*N* = 14) samples. **B** The top five up- and downregulated hallmark pathways detected by GSEA based on the aforementioned comparison. **C** Preferential expression of the immunoglobulin light chain (IGL) over the heavy chain (IGH) in EMM samples.

To further explore the biological features of EMM, we used RNA-sequencing to compare 14 EMM samples with 14 NDMM samples from which 8 samples were paired. In total, we identified 799 significantly deregulated protein coding genes (absolute log2 fold change > 1, Benjamini–Hochberg adjusted p-value < 0.05) among which 290 were downregulated and 509 upregulated (Fig. 4A). Pathway enrichment analysis revealed marked upregulation of pathways connected to cell proliferation and downregulation of pathways connected to immune response in comparison with NDMM (Fig. 4B) as well as in unrelated RRMM samples (Supplementary Fig. 5). Among the most significantly upregulated genes, we identified common biomarkers in human cancers: *STC2*, *CACNA1C*, and *GAGE2A* [21–23]. The most downregulated genes were *FCGR2B*, which was previously connected to PC persistence in BM and higher susceptibility to apoptosis [24], *LYZ*, and *ITPRIP*. In addition, we observed decreased expression of deubiquitinase gene *OTUD1*, which we recently found associated with worse prognosis in MM patients [25]. Importantly, we detected lower expression of *CXCR4*, which encodes a key molecule for PCs homing to the BM [2], and integrin gene *ITGA6*, the downregulation of which was recently connected to progression from MM to plasma cell leukemia [26]. Similarly, expression of *CKS1B* was increased probably due to presence of 1q21 gains. Notably, expression of interleukin 6 (IL6), a potent growth factor for PCs [18], was upregulated, suggesting autocrine regulation of EMM tumor growth. Furthermore, we observed higher production of light chains (IGL) compared to heavy chains in almost all samples (Fig. 4C). Congruent with our previous research, we detected significantly higher expression of important epigenetic mediators in EMM compared to MM: a histone methyltransferase (*EZH2*) and two out of three human DNA methyltransferases (*DNMT1* and *DNMT3B*) [27]. Furthermore, we detected 33 downregulated and 62 upregulated lncRNAs. Among these, we identified upregulation of several lncRNAs previously connected to tumor progression, including *EPIC1* [28], *CASC9* [29], and *ZFPM2-AS1* [30].

### Therapeutically relevant targets are downregulated in EMM cells

**Fig. 5:**
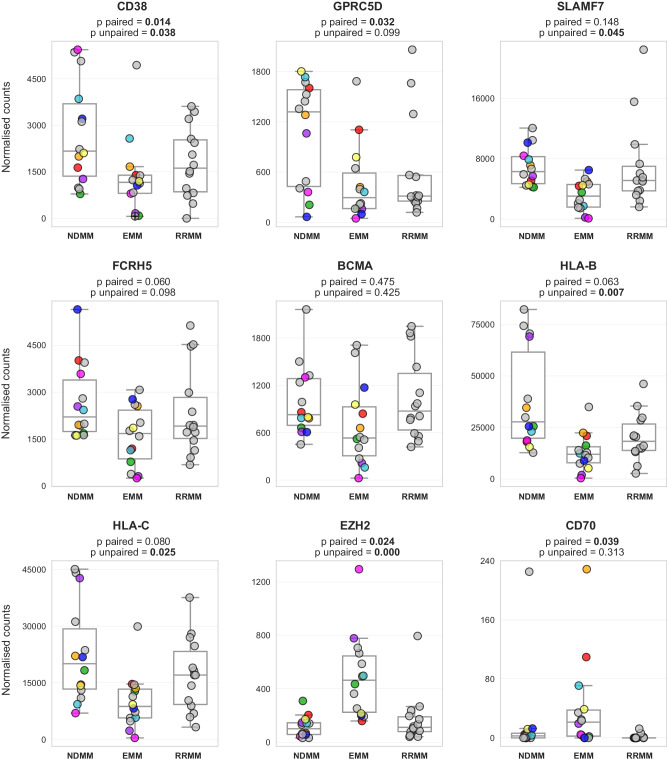
Expression level of clinically important molecules. Boxplots based on the normalized expression level of select therapeutically relevant targets, HLA-B/C, and potentially new targets EZH2 and CD70. Significance between NDMM and EMM samples is stated for both unpaired (14 vs. 14 samples) and paired settings (8 samples; depicted in color). Count normalization (used for visualization) and significance were obtained using DESeq2. RRMM samples (*N* = 14) represent unrelated advanced MM patients without EMM. A grey dot with a black cross represents a sample with very low expression of CD38 from Supplementary Fig. 8.

As the efficacy of anti-myeloma therapy, including novel immunotherapy, is suboptimal in EMM [8, 27, 31–33], we focused on the expression of immunotherapeutic targets, which could predict treatment efficacy. To compare the expression of key molecular targets for MM treatment, we used the same RNA-seq data as in the previous experiments (Fig. 4). We also performed differential expression analysis using only paired samples (*N* = 8) to better capture the longitudinal changes. In addition, we included 14 unrelated RRMM samples for comparison with advanced MM disease without EMM. We observed significantly decreased expression of important molecular targets that are currently in use for MM treatment (*CD38, SLAMF7*, and *GPRC5D*) and a strong trend of lower expression for *FCRH5* (Fig. 5). Notably, no deregulation was observed for *BCMA*. Intriguingly, our data show high expression of *EZH2* and *CD70*, which are promising targets in other malignancies [34–36]. In addition, we observed decreased expression of MHC-I molecules HLA-B and HLA-C in EMM cells, a phenomenon previously associated with a worse response to immunotherapy in other types of cancer [37]. However, this might not be clinically relevant as these genes remained between the top expressed genes (Supplementary Fig. 6).

### CD8+ T and NK cells are predominant immune effector cells in the EMM tumor microenvironment

**Fig. 6:**
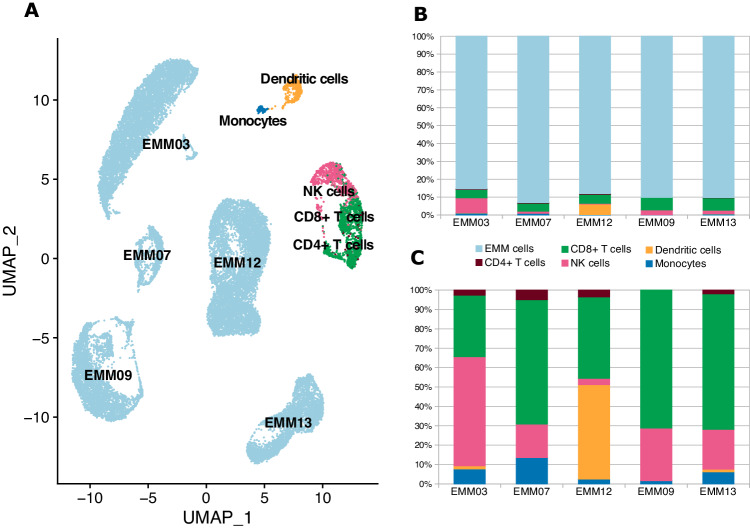
Composition of the EMM tumor microenvironment. **A** UMAP combining cells from five EMM samples. **B **Stacked bar plot describing the composition in whole samples. **C** Bar plot focusing only on immune cells, excluding EMM cells.

To better understand the biology and composition of EMM tumors, we used, for the first time, scRNA-seq to sequence soft tissue tumors in five EMM patients. We achieved a median of 3916 cells per sample. The majority of cells were identified as aberrant PCs (median 90.5%) and the rest were mainly CD8 + T cells, NK cells, monocytes, and CD4+ T cells (median 7%, 1.8%, 0.5%, and 0.2%, respectively; Fig. 6). Importantly, we observed CD8+ T and NK cells as the most abundant immune cells with comparable proportions in flow cytometry (Supplementary Fig. 7). In addition, we observed high heterogeneity of EMM cells (Fig. 6), which could be further demonstrated on typical PC molecules; for example, the EMM cells of one patient virtually lost expression of CD38 and tumor cells from another patient had very low CD138 expression. Importantly, these data are congruent with bulk RNA-seq and flow cytometry data (Supplementary Fig. 8).

## Discussion

**Fig. 7:**
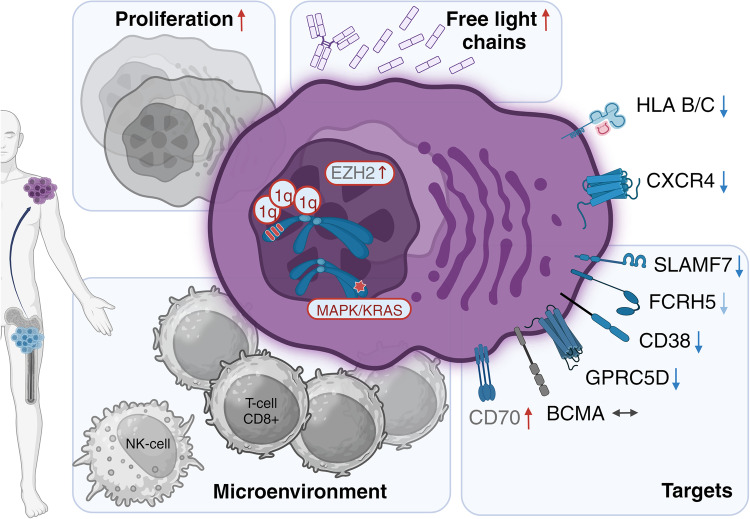
Schematic overview of our key findings on EMM tumors. EMM cells have a higher frequency of 1q21/gain and mutations in the MAPK pathway, mainly in KRAS. Arrows indicate a higher level of proliferation and free light chains and expression of EZH2 and lower expression of CXCR4, HLA B and C, and many important therapeutic targets compared to MM. Finally, the EMM tumor microenvironment is mainly composed of CD8+ and NK cells.

The molecular mechanisms mediating EMM development and resistance are still poorly understood. In contrast to MM, virtually no NGS data describe EMM tumors. The need for such data has become even more evident as the incidence of EMM has dramatically increased along with the prolonged survival of MM patients. Indeed, recent clinical trials report EMM in approximately 40% of RRMM patients [8, 9]. In this study, we present the largest and most comprehensive NGS study of EMM tumor cells to date, describing the key molecular characteristics of EMM, including the expression levels of important therapeutic targets (Fig. 7). We also provide the first insights into the composition of the EMM tumor microenvironment using scRNA-seq data.

Over a decade ago, Billecke et al. reported t(4;14), del(13q), and del(17p) as the most frequent cytogenetic abnormalities, found in 37%, 35%, and 32% of 19 EMM patients, respectively [13]. However, the inclusion of 1q21 in the FISH panel revealed an even higher frequency of this abnormality (13/18, 72%) [14]. Nevertheless, only around 15% of NDMM patients with 1q21 gain/amp progress to EMM [38, 39]. Importantly, analysis using genetic mutation panels identified RAS/BRAF mutations in 73% (8/11) [15] and MAPK pathway mutations in 100% of EMM samples (6/6) [16]. In our study, we detected by FISH and/or WES 1q21 gain/amp in 12/14 EMM samples and identified at least one mutated gene in the MAPK signaling pathway in almost all EMM samples (13/14, 93%), with *KRAS* as the most frequently mutated gene (10/14, 71%).

Based on our and previously published data we hypothesize that EMM development is mostly driven by the interplay between 1q21 gain/amp and mutations in the MAPK pathway, as we detected this combination in 11/14 patients (79%). In particular, *KRAS* was mutated at a markedly higher frequency (71%) than previously reported in NDMM (21.8%) [40] or RRMM (19%) [41]. Furthermore, these mutations could often be detected already at the time of diagnosis. Therefore, we tested our hypothesis on patients from a CoMMpass dataset (*N* = 699) and showed that patients with mutated *KRAS* and 1q21 gain/amp have a significantly higher risk of EMM development (HR = 2.4, *p* = 0.011). This may be particularly important in the near future when such information could easily be available with the implementation of diagnostic gene panels in routine clinical practice [42, 43] and may influence the choice of patient-tailored therapy. Despite the exact molecular mechanisms still remaining elusive, we speculate that both mutations in KRAS and gain/amp of 1q21 can synergistically over-activate the MAPK pathway through constitutively active RAS signaling [44] and over-expression of CKS1B [45], respectively, resulting in increased cell proliferation, dissemination, and resistance [46]. In fact, CKS1B was the only previously suggested myeloma driver on 1q21 [47] that was significantly upregulated in our analysis.

The effect of any routinely used anti-myeloma drugs on the increased incidence of EMM development remains unclear. Higher incidence of secondary EMM in the era of novel drugs is mainly explained by prolonged survival and improved imaging techniques [1]. No evidence of increased risk of EMM was observed for lenalidomide-bortezomib combinations [48], nevertheless this question has been raised dominantly after the introduction of CD38 targeted therapy. Despite limited numbers we detected relatively high occurrence of CD38 deletion in 4/14 of EMM samples in total and in 2/7 of EMM samples pretreated with anti-CD38 mAbs. Regarding patients who underwent ASCT (*N* = 9), we observed relatively low contribution of HD melphalan on overall mutational burden.

Though genomic changes have the potential to provide insights into the mutational events presumably leading to EMM development, especially when paired NDMM samples are available, they fail to offer sufficient understanding of the actual phenotype of EMM cells. Unfortunately, our current knowledge of gene expression in EMM is limited to a gene expression profiling experiment involving six patients conducted approximately 20 years ago [17], four single-cell EMM samples obtained from ascites and pleural effusions [18], and a recent brief communication by our group focusing primarily on CD38 expression in five paired EMM samples [27]. Therefore, this study represents the largest transcriptomic analysis of soft tissue EMM samples to date.

We revealed expression pattern towards higher proliferation rate of EMM cells, as previously demonstrated by a high proliferation index determined during histological examination [49]. The increased proliferation is likely stimulated in an autocrine manner through the production of IL6, which is not typically produced by PCs or MM cells [18]. Consistent with a case report describing tumor cells in a patient with skin-related EMM, we observed decreased expression of *CXCR4*, the protein product of which is responsible for the normal homing of PCs to BM [50].

Intriguingly, based on experiments with mouse models, Roccaro et al. suggested that higher expression of CXCR4 mediates EMM development through the acquisition of an epithelial-to-mesenchymal (ETM) phenotype [51]. As our human samples likely do not represent the ETM phenotype responsible for disease spread, but rather cells from well-established solid-like tumors, we cannot exclude the possibility of higher CXCR4 production in the early stages of EMM development.

Previously published data and current results show upregulation of chromatin modifiers EZH2, DNMT1 and DNMT3B in the EMM samples [27]. This suggests that changes in gene expression during the EMM stage could be regulated by epigenetic mechanisms. Importantly, inhibition of EZH2 is currently used in the treatment for other malignancies, including follicular lymphoma; and in MM it can lead to re-expression of CD38 [34, 52].

Immunotherapy is the most promising therapeutic modality for MM patients. Three naked mAbs are currently FDA approved: daratumumab and isatuximab targeting CD38, and elotozumab targeting SLAMF7 [53–55]. There are also two FDA-approved CAR-T products, both targeting BCMA: cilta-cel and ide-cel [56, 57]. Finally, there are also two FDA-approved bispecific antibodies (bsAbs) targeting BCMA (teclistamab and elranatamab) and one targeting GPRC5D (talquetamab) [8, 33, 58]. Many others are under clinical development, such as cevostamab targeting FCRH5 [59]. The efficacy of these agents is dependent on the patient´s immune effector cells, as well as on the expression level of the targets on the surface of malignant PCs.

Our data revealed decreased expression of CD38, SLAMF7, GPRC5D, and FCRH5 in EMM cells but no changes in expression for BCMA. We also observed lower expression of MHC-I genes on EMM cells (*HLA-B*, *HLA-C*), which may be associated with reduced efficacy of cancer immunotherapies due to decreased recognition of malignant cells by effector T cells [37, 60]. Many recent studies reported lower efficacy of modern immunotherapy in patients with EMM relapse for daratumumab, teclistamab, talquetamab, and others [8, 27, 33]. On the other hand, we observed elevated CD70 expression in EMM. This could potentially represent a way to activate the immune system by blocking the CD27-CD70 axis [36]. This concept has already been tested in trials for solid tumors using CAR-T agents.

For further elucidation of immunotherapy efficacy, it is crucial to study the tumor microenvironment of EMM, which has not been described to date. Previously, Ryu et al. reported the presence of several immune populations from ascites (*n* = 2) and pleural effusion (*n* = 1) samples from patients with EMM [18]. However, these samples do not represent true soft tissue EMM tumors. Therefore, we provide the first insight into EMM tumors at single-cell resolution, showing that the majority of the tumor mass is composed of aberrant PCs, and approximately 10% of the tumor is then formed by immune cells, mainly CD8+ T cells and NK cells. These immune cell subsets might be vital for adequate cell killing by novel immunotherapeutic agents. Nevertheless, low proportion of immune cells in EMM tumors prevents detailed characterization of these populations and represent a limitation in our scRNA data. An example of the most effective therapy for EMM to date seems to be the combination of teclistamab and talquetamab targeting two different antigens [9].

Despite the limited number of EMM samples, our study stands as the most comprehensive and largest NGS study of EMM to date, given the difficulty to obtain bioptable EMM samples. Our findings suggest that the development of EMM from MM cannot be attributed to a single mutation event, but rather a combination of several genetic, and possibly epigenetic, changes. Importantly, these changes are usually not present at diagnosis, but acquired later during the disease, as demonstrated by our unprecedented longitudinal data. Furthermore, we describe significant alterations in the expression profile of EMM cells compared to MM cells in the BM, including clinically important targets and CXCR4. Additionally, our study provides the first insights into the EMM tumor microenvironment.

### Supplementary information

Supplementary Material

Supplementary Tables

## Data Availability

The datasets generated during the current study are available in the European Genome-phenome Archive (EGA) repository under the accession numbers: EGAD50000000051- EGAD50000000053.
