# Supplementary material for: Beyond the marrow: insights from comprehensive next-generation sequencing of extramedullary multiple myeloma tumors
Source: Leukemia. 2024 Mar 16;38(6):1323–33. doi: 10.1038/s41375-024-02206-w (PMC11147761; doi:10.1038/s41375-024-02206-w)
Supplement: Supplementary file 1 — Supplementary Material [file 41375_2024_2206_MOESM1_ESM.pdf]

SUPPLEMENTARY MATERIAL

Beyond the Marrow: Insights from Comprehensive Next-Generation Sequencing of Extramedullary Multiple Myeloma Tumors

Jelinek T.[1,2]*, Zihala D.[1,2]*, Sevcikova T.[1,2]*, Anilkumar Sithara A.[1,2,3], Kapustova V.[1,2], Sahinbegovic H.[1,2,3], Venglar O.[1,2,3], Muronova L.[1,2], Broskevicova L.[1,2], Nenarokov S. [1,2], Bilek D.[3], Popkova T.[1,2], Plonkova H.[1], Vrana J.[1,2], Zidlik V.[4], Hurnik P.[4,] Havel M.[5], Hrdinka M.[1,2], Chyra Z.[1,2], Stracquadanio G.[6], Simicek M.[1,2], Hajek R.[1,2]

** These authors contributed equally and are considered joint first authors*

Corresponding author:

Prof. Roman Hajek, MD, PhD

Department of Hematooncology

University Hospital Ostrava

17. listopadu 1790/5, 708 52 Ostrava, Czech Republic

Phone: +420 597 37 2151, Fax: +420 597 37 2092

Email: roman.hajek@fno.cz

1 Department of Hematooncology, Faculty of Medicine, University of Ostrava, Ostrava, Czech Republic

2 Department of Hematooncology , University Hospital Ostrava, Ostrava, Czech Republic

3 Faculty of Science, University of Ostrava, Ostrava, Czech Republic

4 Department of Pathology, University Hospital Ostrava, Ostrava, Czech Republic

5 Department of Nuclear Medicine, University Hospital Ostrava, Ostrava, Czech Republic and Department of Imaging Methods, Faculty of Medicine, University of Ostrava, Ostrava, Czech Republic

6 School of Biological Sciences, The University of Edinburgh, Edinburgh EH9 3BF, UK

Table of Contents

Table of Contents 2
Supplementary methods 2
Supplementary figures...9

Supplementary methods

Fluorescence-activated flow sorting

Samples from the time of diagnosis paired with EMM samples were obtained from the Biobank as frozen bone marrow mononuclear cells (BMMC) or frozen magnetic-activated cell sorted (MACS) plasma cells using CD138 magnetic beads (Miltenyi Biotec, Germany). BMMC samples, and some MACS samples with sufficient numbers of cells, were subsequently FACS sorted as described in the section "FACS" in the main text. T-lymphocytes were used as normal cell population for exome sequencing and were sorted using CD3 marker from peripheral blood or bone marrow.

Whole Exome Sequencing data analysis

The raw reads were aligned to the human reference genome GRCh38 using BWA MEM[1] v0.7.17 and sorted and indexed with Sambamba[2] v0.8.2 . The quality of the raw data and alignments was investigated using Picard[3] v2.9.2 and MultiQC[4] v1.9. Somatic single nucleotide variants (SNVs) and short insertions or deletions (INDELs) were identified using GATK[5] v4.1.4.1 Mutect2[6], Strelka[7] v2.9.10, Manta[8] v1.6.0 and Varscan[9] v2.4.3 and were annotated with vcf2maf[10] v1.6.21 algorithm. Variants detected by at least two variant callers with Variant Allele Frequency > 0.05 were considered for downstream analysis. Furthermore, silent mutations, RNA mutations or mutations in Intronic or Intergenic regions were filtered out. Selected mutations were visualized as Oncoplot using Maftools[11] algorithm. The Copy number Aberrations(CNA) were detected using Sequenza[12] v3.0.0 with a default ploidy between 2 and 2.8. Cancer cell fraction (CCF) was estimated using PyClone[13] v0.13.1 utilizing the filtered mutations and CNAs detected by Sequenza. Visualization of the

WES results was performed combining Maftools and Inkscape tool for vector graphics. Mutational signatures were investigated using mmsig[14] algorithm with cosine similarity threshold of 0.05. The stricter threshold was chosen since the default threshold of 0.01 resulted in overfitting of the data with SBS-MM1 signature corresponding to Melphalan exposure in one patient sample at diagnosis without any reported Melphalan exposure history.

Transcriptome data analysis

The raw fastq files were trimmed for adapter and low-quality reads using TrimGalore v0.6.6, a wrapper of the Cutadapt[15] program and SortMeRNA[16] v4.2.0 was used for filtering out rRNA reads. Furthermore, STAR[17] aligner v2.7.7a and Qualimap[18] v2.2.2-dev were used for additional quality control. Reads passing the quality check was further subjected to transcript quantification using Salmon[19] v1.4.0. Differential gene expression analyses were performed with DESeq2[20] v1.30.0. Significant genes were selected based on following criteria: with Benjamini-Hochberg adjusted p-value < 0.05 and absolute value of Log2 Fold change > 1. The analysis of fusion transcripts was performed using three different algorithms namely Arriba[21] v2.1.0, FusionCatcher[22] v1.33 and Star-fusion[23] v1.6.0. The TRUST4[24] v1.0.5.1 algorithm for immune repertoire reconstruction from bulk RNA-seq data was used to determine the most abundant Immunoglobulin heavy and light chains. Corresponding abundance of the transcripts in TPM were estimated from Salmon counts and pairwise comparison of IG was performed in EMM samples. Finally, data were visualized using the R and Inkscape tool for vector graphics.

Single cell transcriptomic data analysis

Single cell RNA-seq (scRNAseq) data from EMM tumors from five patients were processed using 10x Genomics Cell Ranger[25] 7.1.0. EMM tumor sample EMM09 was sequenced twice in separate batches and was merged for downstream analyses. The raw reads were aligned to human reference genome GRCh38 followed by filtering and barcode counting. The filtered feature barcode matrix was then processed with the R package Seurat R toolkit[26]. In addition to the conventional filtering of low-quality cells

or empty droplets with Seurat we used SoupX[27] tool for removing ambient RNA contamination. Furthermore, DoubletFinder[28] algorithm was integrated for detecting doublets. The count data was then log-normalized and 2,000 highly variable features were identified. Principal component analysis was performed on scaled data with previously determined variable features. The first 15 components were selected for dimensional reduction and then clustering was performed. The differentially expressed genes in each cluster were identified with the seurat function FindAllMarkers and the canonical markers were used to match the clusters to known immune cell types. Additionally, the algorithm SingleR[29] together with the celldex package was used for automated annotation of the identified clusters. The major clusters of EMM cells from each sample were identified manually with the identified set of markers and the other immune cell types were identified utilizing SingleR with Novershtern hematopoietic data[30] and Monaco Immune data[31] references from celldex.

Flow cytometric analysis of EMM tumor microenvironment

Flow cytometry of basic immune cell subsets from EMM tumor cell suspensions was performed with the Euroflow lymphoproliferative disorder screening tube panel[32]. The material was processed according to Euroflow standard operating protocols for sample preparation[33]. Stained samples were acquired on a BD Canto II equipped with 405 nm, 488 nm, and 633 nm lasers. Data were analyzed by Infinicyt software version 2.0. Gating was performed on events free of debris and doublets. T cells were defined as CD3+ CD19- CD45+ SSClow with further subdivision to CD4+ or CD8+ subsets. NK cells were gated as CD3- CD19- CD56+/dim CD45+ SSClow.

Survival analysis

Survival analysis of Multiple Myeloma Research Foundation CoMMpass study (NCT01454297) ("CoMMpass", N=699, IA20) data was performed using univariate and multivariate Cox models and a multivariate Fine-Gray model to evaluate the risk of EMM. 'MMRF_CoMMpass_IA20_PER_PATIENT.tsv',

‘MMRF_CoMMpass_IA20_PER_PATIENT_VIS-IT.tsv’ and ‘MMRF_CoMMpass_IA20_STAND_ALONE_SURVIVAL.tsv’ from clinical datafiles was used to obtain clinical and survival characteristics of patients measured at the first visit. Furthermore ‘Somatic Mutation Files - SNV and INDEL MMRF_CoMMpass_IA20_combined_vcfmerger2_All_ Canonical_NS_Variants_Gene_Mutation_Counts.tsv’ and ‘SeqFISH Files_MMRF_CoMMpass_IA20_exome_gatk_cna_seqFISH.tsv’ files were used to identify somatic mutations and chromosomal aberrations of patients respectively. The dataset was filtered to contain only patients with valid profiling of chromosomal aberrations and mutations. Additional filters were applied to exclude patients with LDH measurements and ISS stage classification unavailable at baseline. Patients with single or multiple plasmacytomas at any visits were identified from the variable ‘ST_NUMBEROFPLASM’ of ‘MMRF_CoMMpass_IA20_PER_PATIENT_VISIT.tsv’ dataset and was used as the indicator of EMM disease. Days until EMM occurrence or survival were estimated from variables ‘VISITDY’ and ‘ttcos’. Patients with KRAS gene mutated were identified from the dataset of somatic mutations filtered for Gene "ENSG00000133703". High-risk CAs defined as gain/amp1q21, del 13q14 and del17p13 with a threshold of 20% for positive detection by Seq-FISH were considered for the analysis. Univariate and multivariate Cox models and multivariate Fine-Gray mode was used to assess the impact of age(<= 65 vs > 65), ISS, LDH level, del 13q14, del 17p13, mutation in KRAS gene, gain/amp 1q21 or a combination of KRAS mutation and gain/amp 1q21 on the occurrence of EMM. We used p=0.05 as a threshold for significance in all analyses. We performed all computations and visualization using R(v4.0.3) and survival(v3.2.11), survminer(v0.4.9), lubridate(v1.7.10), readxl(v1.3.1), and tidyverse(v1.3.1) packages.

1 Fast and accurate short read alignment with Burrows–Wheeler transform | Bioinformatics | Oxford Academic. https://academic.oup.com/bioinformatics/article/25/14/1754/225615 (accessed 13 Feb2024).

2 Tarasov A, Vilella AJ, Cuppen E, Nijman IJ, Prins P. Sambamba: fast processing of NGS alignment formats. *Bioinformatics* 2015; **31**: 2032–2034.

3 Picard Tools - By Broad Institute. https://broadinstitute.github.io/picard/ (accessed 13 Feb2024).

4 MultiQC: summarize analysis results for multiple tools and samples in a single report | Bioinformatics | Oxford Academic. https://academic.oup.com/bioinformatics/article/32/19/3047/2196507 (accessed 13 Feb2024).

5 The Genome Analysis Toolkit: A MapReduce framework for analyzing next-generation DNA sequencing data. https://genome.cshlp.org/content/20/9/1297.short (accessed 13 Feb2024).

6 Benjamin D, Sato T, Cibulskis K, Getz G, Stewart C, Lichtenstein L. Calling Somatic SNVs and Indels with Mutect2. 2019; : 861054.

7 Saunders CT, Wong WSW, Swamy S, Becq J, Murray LJ, Cheetham RK. Strelka: accurate somatic small-variant calling from sequenced tumor–normal sample pairs. *Bioinformatics* 2012; **28**: 1811–1817.

8 Manta: rapid detection of structural variants and indels for germline and cancer sequencing applications | Bioinformatics | Oxford Academic. https://academic.oup.com/bioinformatics/article/32/8/1220/1743909 (accessed 13 Feb2024).

9 VarScan: variant detection in massively parallel sequencing of individual and pooled samples | Bioinformatics | Oxford Academic. https://academic.oup.com/bioinformatics/article/25/17/2283/210190 (accessed 13 Feb2024).

10 mskcc/vcf2maf. 2024.https://github.com/mskcc/vcf2maf (accessed 13 Feb2024).

11 Maftools: efficient and comprehensive analysis of somatic variants in cancer. https://genome.cshlp.org/content/28/11/1747.short (accessed 13 Feb2024).

12 Favero F, Joshi T, Marquard AM, Birkbak NJ, Krzystanek M, Li Q *et al.* Sequenza: allele-specific copy number and mutation profiles from tumor sequencing data. *Ann Oncol* 2015; **26**: 64–70.

13 Roth A, Khattra J, Yap D, Wan A, Laks E, Biele J *et al.* PyClone: statistical inference of clonal population structure in cancer. *Nat Methods* 2014; **11**: 396–398.

14 Rustad EH, Nadeu F, Angelopoulos N, Ziccheddu B, Bolli N, Puente XS *et al.* mmsig: a fitting approach to accurately identify somatic mutational signatures in hematological malignancies. *Commun Biol* 2021; **4**: 1–12.

15 Martin M. Cutadapt removes adapter sequences from high-throughput sequencing reads. *EMBnet.journal* 2011; **17**: 10–12.

16 SortMeRNA: fast and accurate filtering of ribosomal RNAs in metatranscriptomic data | Bioinformatics | Oxford Academic. https://academic.oup.com/bioinformatics/article/28/24/3211/246053 (accessed 13 Feb2024).

17 STAR: ultrafast universal RNA-seq aligner | Bioinformatics | Oxford Academic. https://academic.oup.com/bioinformatics/article/29/1/15/272537 (accessed 13 Feb2024).

18 Qualimap: evaluating next-generation sequencing alignment data | Bioinformatics | Oxford Academic. https://academic.oup.com/bioinformatics/article/28/20/2678/206551 (accessed 13 Feb2024).

19 Patro R, Duggal G, Love MI, Irizarry RA, Kingsford C. Salmon provides fast and bias-aware quantification of transcript expression. *Nat Methods* 2017; **14**: 417–419.

20 Love MI, Huber W, Anders S. Moderated estimation of fold change and dispersion for RNA-seq data with DESeq2. *Genome Biol* 2014; **15**: 550.

21 Burgos L, Tamariz-Amador L-E, Puig N, Cedena M-T, Guerrero C, Jelínek T *et al.* Definition and Clinical Significance of the Monoclonal Gammopathy of Undetermined Significance–Like Phenotype in Patients With Monoclonal Gammopathies. *J Clin Oncol* 2023; **41**: 3019–3031.

22 FusionCatcher – a tool for finding somatic fusion genes in paired-end RNA-sequencing data | bioRxiv. https://www.biorxiv.org/content/10.1101/011650v1 (accessed 13 Feb2024).

23 Haas BJ, Dobin A, Stransky N, Li B, Yang X, Tickle T *et al.* STAR-Fusion: Fast and Accurate Fusion Transcript Detection from RNA-Seq. 2017; : 120295.

24 Song L, Cohen D, Ouyang Z, Cao Y, Hu X, Liu XS. TRUST4: immune repertoire reconstruction from bulk and single-cell RNA-seq data. *Nat Methods* 2021; **18**: 627–630.

25 Zheng GXY, Terry JM, Belgrader P, Ryvkin P, Bent ZW, Wilson R *et al.* Massively parallel digital transcriptional profiling of single cells. *Nat Commun* 2017; **8**: 14049.

26 Hao Y, Hao S, Andersen-Nissen E, Mauck WM, Zheng S, Butler A *et al.* Integrated analysis of multimodal single-cell data. *Cell* 2021; **184**: 3573-3587.e29.

27 Young MD, Behjati S. SoupX removes ambient RNA contamination from droplet-based single-cell RNA sequencing data. *GigaScience* 2020; **9**: giaa151.

28 McGinnis CS, Murrow LM, Gartner ZJ. DoubletFinder: Doublet Detection in Single-Cell RNA Sequencing Data Using Artificial Nearest Neighbors. *Cell Syst* 2019; **8**: 329-337.e4.

29 Aran D, Looney AP, Liu L, Wu E, Fong V, Hsu A *et al.* Reference-based analysis of lung single-cell sequencing reveals a transitional profibrotic macrophage. *Nat Immunol* 2019; **20**: 163–172.

30 Novershtern N, Subramanian A, Lawton LN, Mak RH, Haining WN, McConkey ME *et al.* Densely interconnected transcriptional circuits control cell states in human hematopoiesis. *Cell* 2011; **144**: 296–309.

31 Monaco G, Lee B, Xu W, Mustafah S, Hwang YY, Carré C *et al.* RNA-Seq Signatures Normalized by mRNA Abundance Allow Absolute Deconvolution of Human Immune Cell Types. *Cell Rep* 2019; **26**: 1627-1640.e7.

32 van Dongen JJM, Lhermitte L, Böttcher S, Almeida J, van der Velden VHJ, Flores-Montero J *et al.* EuroFlow antibody panels for standardized n-dimensional flow cytometric immunophenotyping of normal, reactive and malignant leukocytes. *Leukemia* 2012; **26**: 1908–1975.

33 Kalina T, Flores-Montero J, van der Velden VHJ, Martin-Ayuso M, Böttcher S, Ritgen M *et al.* EuroFlow standardization of flow cytometer instrument settings and immunophenotyping protocols. *Leukemia* 2012; **26**: 1986–2010.

Supplementary figures

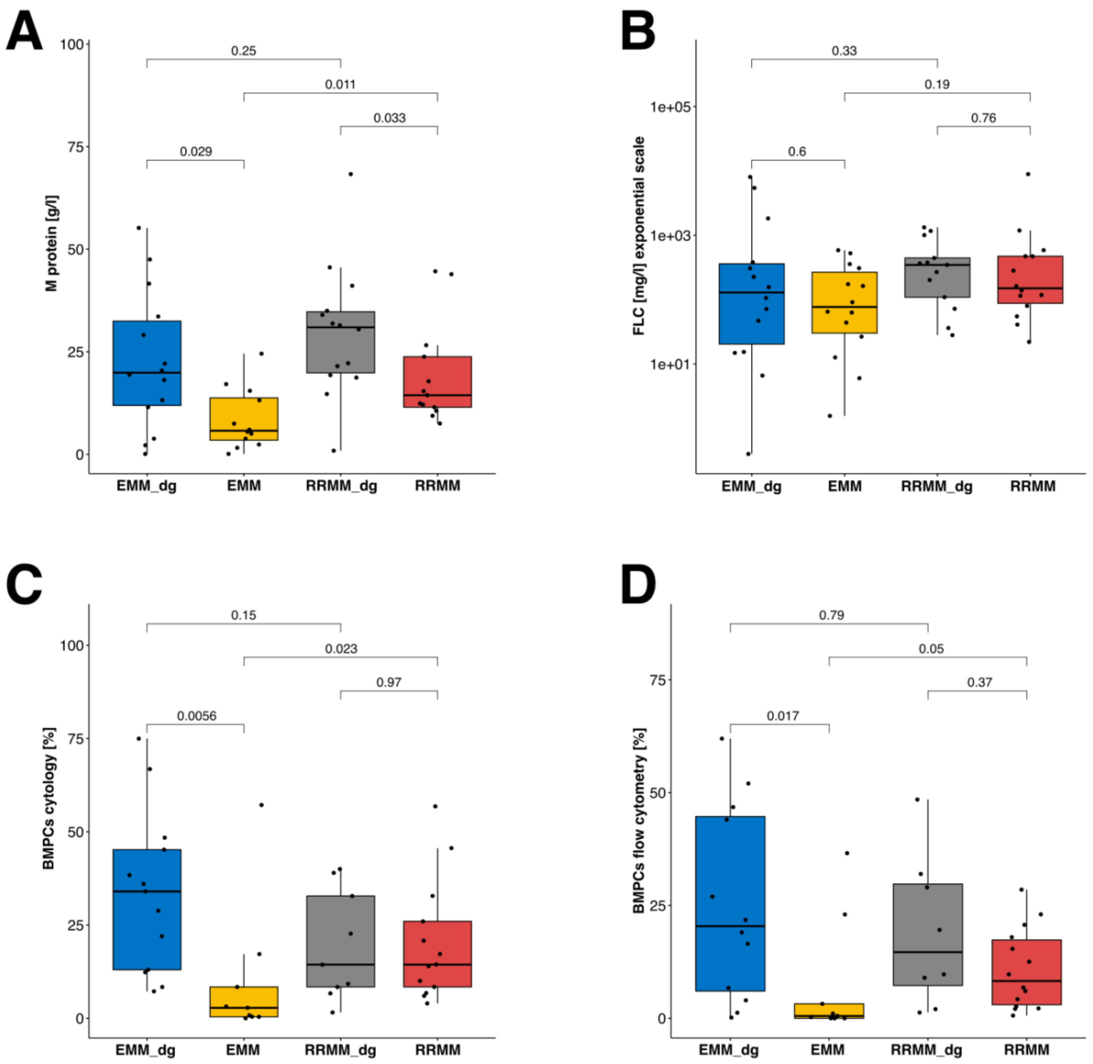

Supplementary Figure 1: Comparison of basic characteristics of EMM and RRMM cohorts: **(A)** M-protein levels; **(B)** FLC levels; **(C)** BMPCs measured by cytology; **(D)** BMPCs measured by flow cytometry. Abbreviations: EMM_dg: EMM patients at diagnosis; EMM: EMM patients at the time of relapse with EMM; RRMM_dg: relapse/refractory patients at diagnosis; RRMM: relapse/refractory patients at relapse. Statistical significance was inferred using nonparametric Mann-Whitney U test.

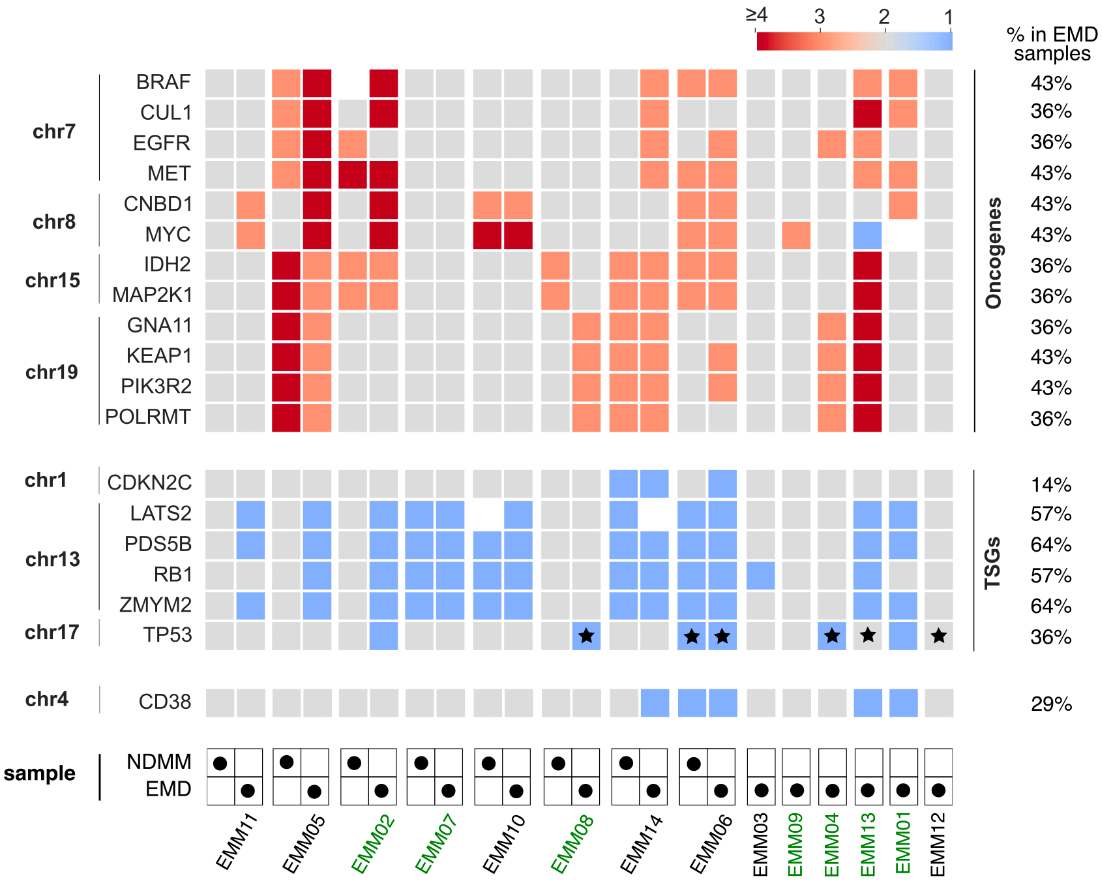

Supplementary Figure 2: Most frequently amplified oncogenes and frequently deleted tumor suppressor genes (TSGs) and *CD38* in EMM cells. Paired NDMM and EMM samples are grouped together. Patients that underwent anti-CD38 treatment are highlighted by green color.

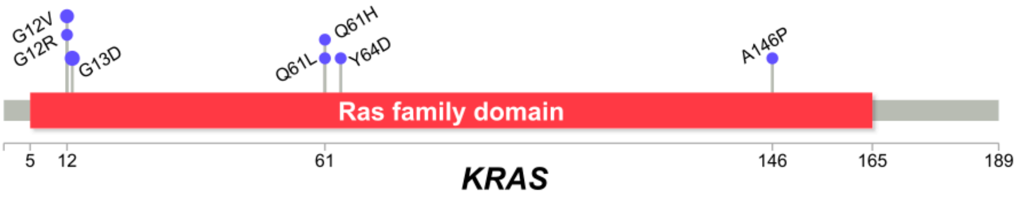

Supplementary Figure 3: All mutations in the KRAS gene detected in EMM samples. Larger dot indicates a mutation detected in two different samples.

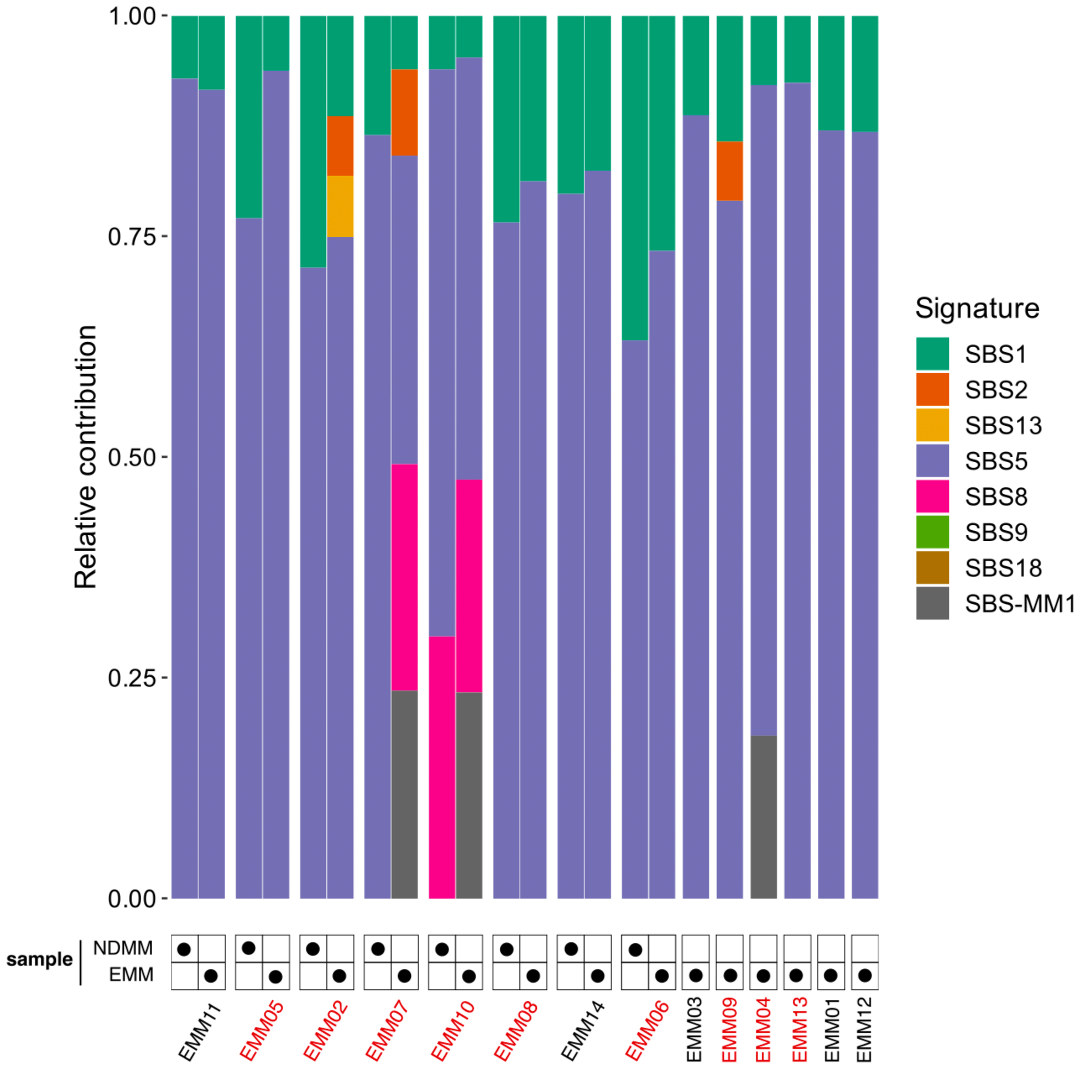

Supplementary Figure 4: Relative contribution of different mutational signatures to the overall mutational burden detected by mmsig software (details are described in Supp. Methods). Patients that underwent ASCT are highlighted by red color.

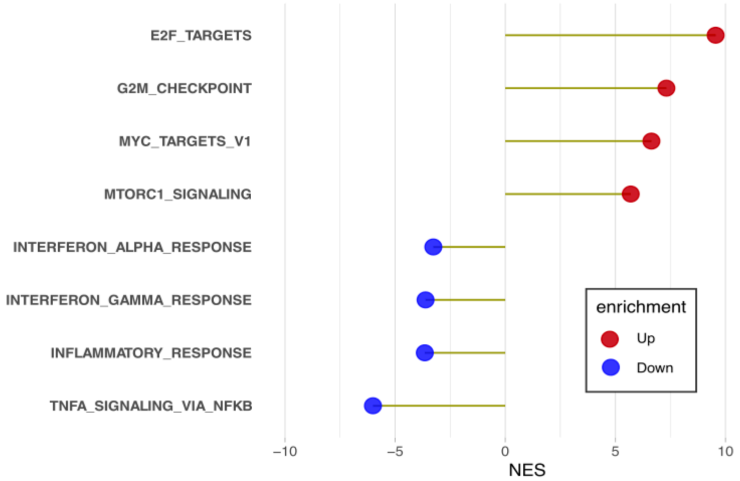

Supplementary Figure 5: Pathway enrichment analysis based on comparison of 14 EMM and 14 unrelated RRMM samples. Red and blue colors depict pathways that are up-regulated and down-regulated in EMM, respectively.

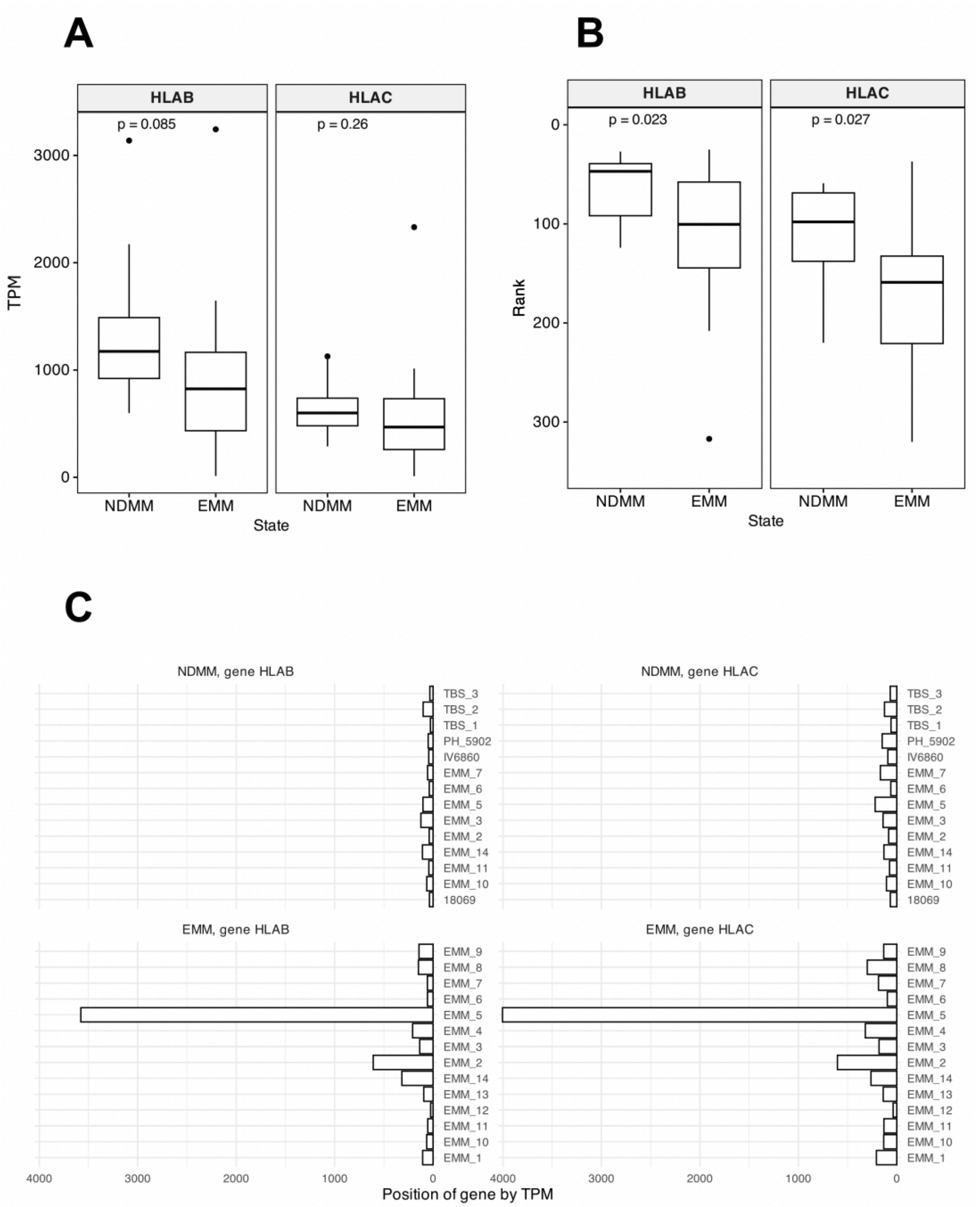

Supplementary Figure 6: Level of expression of HLA-B/C genes represented as A) TPM; Rank among all expressed genes based on TPM visualized as B) a boxplot and C) a bar plot. Statistical significance was evaluated using Mann-Whitney U test.

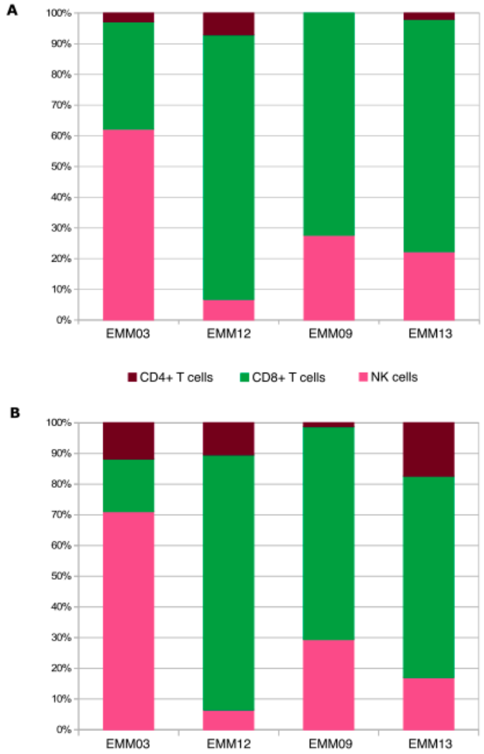

Supplementary Figure 7: CD4+ T cells, CD8+ T cells and NK cells detected in comparable proportions by **(A)** scRNA-seq and **(B)** flow cytometry (available for 4/5 patients).

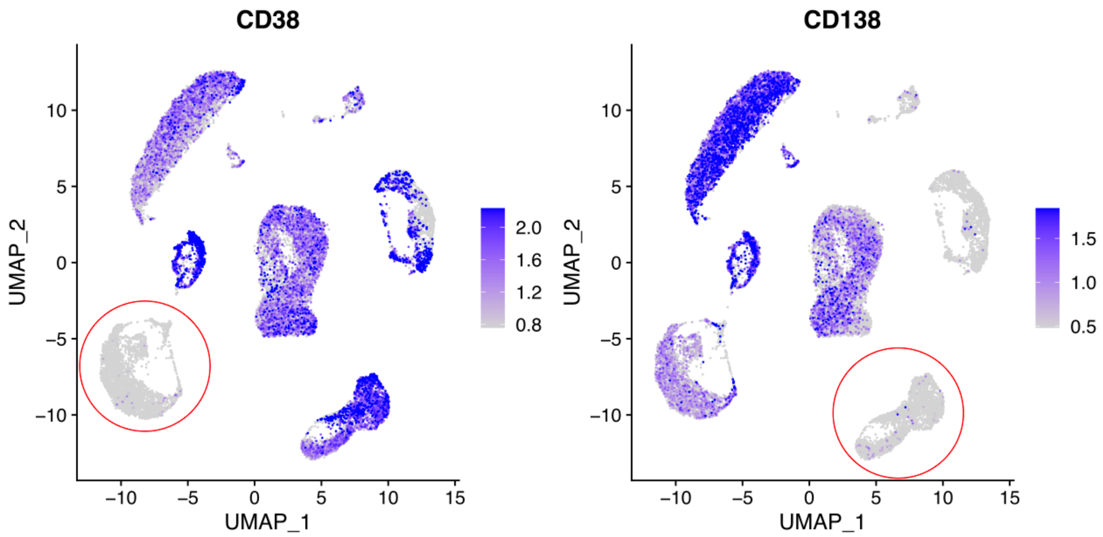

Supplementary Figure 8: Loss of expression of typical markers CD38 (left; EMM09) and CD138 (right; EMM13) of EMM tumor cells of selected patients. These results are congruent with observations form bulk RNA (sample with low expression of CD38 is highlighted in the Fig. 5 with a black cross) and flow cytometry analyses.
