# Supplementary material for: Beyond the marrow: insights from comprehensive next-generation sequencing of extramedullary multiple myeloma tumors
Source: Leukemia. 2024 Mar 16;38(6):1323–33. doi: 10.1038/s41375-024-02206-w (PMC11147761; doi:10.1038/s41375-024-02206-w)
Supplement: Supplementary file 2 — Supplementary Tables [file 41375_2024_2206_MOESM2_ESM.pdf]

Supplementary Table 1: Clinical characteristic of EMM patients

Patient	Sex	ISS stage	Ig type	Light chain type	M protein level at diagnosis (g/l)	M protein level at EMM (g/l)	FLC at diagnosis (mg/l)	FLC at EMM (mg/l)	BMPCs at diagnosis measured by flow cytometry (%)	BMPCs at EMM measured by flow cytometry (%)	BMPCs at diagnosis measured by cytology (%)	BMPCs at EMM measured by cytology (%)	LDH at diagnosis (µkat/l)	LDH at EMM (µkat/l)
EMM01	F	2	IgA	kappa	22.1	15.5	156.7	526	52	NA	48.4	NA	NA	3.15
EMM02	M	2	IgA	lambda	2.2	NA	8073	163	NA	NA	34	NA	3.2	8.6
EMM03	M	1	IgG	kappa	3.8	3.8	47	44	1.2	0	7.2	0	2.8	3.44
EMM04	M	2	IgG	lambda	41.6	6	226	357	NA	0	NA	0.8	2.2	5.69
EMM05	M	1	IgG	Kappa	29.1	7.48	0.4	12.64	44	NA	36	NA	2.6	10.8
EMM06	M	1	IgG	kappa	13.2	5.06	71.5	63	4	NA	8.4	NA	2.6	4.8
EMM07	M	2	IgA	kappa	18.1	1.58	1832	585	16.5	3.2	45.2	8.4	5.3	25.6
EMM08	F	2	IgG	kappa	20.4	17.12	106	65	21.8	0.3	22	3.2	2.87	6.2
EMM09	F	1	IgA	kappa	19.4	24.54	14.88	26.6	27	36.6	38.4	57.2	4.32	2.84
EMM10	M	3	IgA	kappa	55.2	0.1	15.4	1.56	62	NA	75	NA	7.5	2.72
EMM11	M	3	IgA	lambda	33.6	13.18	6.6	6	19	23	28.8	17.2	2.34	4.72
EMM12	M	2	FLC	kappa	0.1	NA	307	91	6.7	0.5	12.3	0.4	2.06	4.1
EMM13	M	1	IgG	lambda	11.5	2.4	381	308	0.15	0	13	0.4	3.73	3.41
EMM14	F	3	IgG	lambda	47.5	5.5	5450	174	46.8	1	66.8	2.8	8.27	4.35

Supplementary Table 2: Treatment summary of EMM cohort prior sample collection

Patient	alkylators	ASCT	bortezomib	ixazomib	carfilzomib	thalidomide	lenalidomide	pomalidomide	daratumumab/isatuximab	others	Number of previous lines of therapy before EMM/relapse sampling
EMM01	✓				✓	✓	✓	✓	✓	ibrutinib	6
EMM02	✓	✓	✓	✓			✓		✓		3
EMM03			✓								1
EMM04	✓	✓	✓	✓		✓	✓	✓	✓		3
EMM05	✓	✓	✓	✓		✓	✓	✓		panobinostat	5
EMM06	✓	✓	✓		✓	✓	✓	✓			4
EMM07	✓	✓	✓		✓	✓	✓		✓		5
EMM08	✓	✓	✓			✓	✓		✓		2
EMM09	✓	✓	✓			✓	✓	✓	✓		3
EMM10	✓	✓	✓		✓	✓	✓				4
EMM11	✓		✓			✓	✓				2
EMM12			✓				✓				1
EMM13	✓	✓	✓						✓		1
EMM14			✓			✓	✓				1

Supplementary Table 3: Clinical characteristic of unpaired NDMM and RRMM coho

**Cohort**	NDMM (N=6)	RRMM (N=14)
Gender, No. (%)		
Man	4 (67)	6 (43)
Woman	2 (33)	8 (57)
ISS stage, No. (%)		
I	4 (67)	6 (43)
II	1 (17)	3 (21)
III	1 (17)	5 (36)
Cytogenetics, No. (%)		
Standard risk	5 (83)	8 (57)
High risk	1 (17)	6 (43)
LDH, Median (min-max) [µkat/l]	2.7 (1.7-4.6)	3.5 (1.1-4.8)
NA	1	
Ig type, No. (%)		
IgG	3 (50)	12 (86)
IgA	2 (33)	2 (14)
LC only	0 (0)	0 (0)
NA	1 (17)	0 (0)
Light chain type, No. (%)		
Kappa	2 (33)	11 (79)
Lambda	4 (67)	3 (21)
M protein, Median (min-max) [g/l]	40.7 (35.0-41.1)	14.4 (07.5-44.6)
NA	1	0
FLC, Median (min-max) [mg/l]	283 (129-500)	150 (22-8927)
NA	1	1
% BMPCs, Median (min-max) (flow)	9.4 (3.0-40.6)	8.3 (0.6-28.5)
% BMPCs, Median (min-max) (cytology)	52.8 (5.2-58.0)	14.4 (4.0-56.8)
NA	1	1

Supplementary Table 4: Comphrehensive annotation of mutations from Figure 2B

Hugo_Symbol	Chromosome	Start_Position	Patient	sample	HGVSc	HGVSp	HGVSp_Short	COSMIC	dbSNP_RS	CNt	n_alleles_with_mutation	n_allele_without_mutation	proportion_of_mutated_allele	CCF	VAF_from_RNAseq	total_reads_rnaseq
KRAS	chr12	25227341	EMM01	EMM	c.183A>T	p.Gln61His	p.Q61H	COSV55499223	rs17851045	2	1	1	0.5	1	0.5	107
STAG2	chrX	124037543	EMM01	EMM	c.305G>C	p.Trp102Ser	p.W102S	NA	novel	1	1	0	1	1	1	109
KRAS	chr12	25245347	EMM02	EMM	c.38G>A	p.Gly13Asp	p.G13D	COSV55497388	rs112445441	8	6	2	0.8	1	0.8	282
NRAS	chr1	114713907	EMM03	EMM	c.183A>T	p.Gln61His	p.Q61H	COSV54736991	rs121913255	1	1	0	1	1	1	240
KRAS	chr12	25245350	EMM04	EMM	c.35G>T	p.Gly12Val	p.G12V	COSV55497419	rs121913529	2	1	1	0.5	1	0.5	39
TP53	chr17	7674188	EMM04	EMM	c.775G>T	p.Asp259Tyr	p.D259Y	COSV52675732	rs1567548929	1	1	0	1	1	1	152
EGFR	chr7	55205537	EMM05	EMM	c.3553G>T	p.Gly1185Cys	p.G1185C	COSV51827170	NA	4	3	1	0.8	0.9	0	0
KRAS	chr12	25245347	EMM05	EMM	c.38G>A	p.Gly13Asp	p.G13D	COSV55497388	rs112445441	8	7	1	0.9	1	0.9	19
NF1	chr17	31235939	EMM05	EMM	c.3892C>T	p.Gln1298Ter	p.Q1298*	NA	NA	2	1	1	0.5	1	0	3
SMC1A	chrX	53415029	EMM05	EMM	c.250T>C	p.Tyr84His	p.Y84H	NA	novel	2	1	1	0.5	1	1	9
SMC1A	chrX	53415029	EMM05	NDMM	c.250T>C	p.Tyr84His	p.Y84H	NA	novel	3	2	1	0.7	0.9	0.9	28
ATM	chr11	108302969	EMM06	EMM	c.5441del	p.Leu1814TrpfsTer14	p.L1814Wfs*14	NA	novel	3	2	1	0.7	1	0.2	20
FLT3	chr13	28049751	EMM06	EMM	c.766A>G	p.Thr256Ala	p.T256A	NA	novel	1	1	0	1	1	0	0
FLT3	chr13	28070603	EMM06	EMM	c.52dup	p.Ser18PhefsTer24	p.S18Ffs*24	NA	novel	1	1	0	1	1	0	0
MSH6	chr2	47803501	EMM06	EMM	c.3261del	p.Phe1088SerfsTer2	p.F1088Sfs*2	COSV99029757	rs267608078	1	1	0	1	1	0.9	26
NF1	chr17	31219018	EMM06	EMM	c.1541_1542del	p.Gln514ArgfsTer43	p.Q514Rfs*43	COSV62194613	rs267606600	1	1	0	1	1	1	33
TP53	chr17	7676047	EMM06	EMM	c.322G>A	p.Gly108Ser	p.G108S	COSV52862450	rs587782461	1	1	0	1	1	1	118
TP53	chr17	7676077	EMM06	EMM	c.292C>T	p.Pro98Ser	p.P98S	COSV52787732	rs1597374015	1	1	0	1	1	1	78
FLT3	chr13	28070603	EMM06	NDMM	c.52dup	p.Ser18PhefsTer24	p.S18Ffs*24	NA	novel	1	1	0	1	1	0	0
KRAS	chr12	25245345	EMM06	NDMM	c.40G>A	p.Val14Ile	p.V14I	COSV55501342	rs104894365	2	2	0	1	1	1	25
MSH6	chr2	47803501	EMM06	NDMM	c.3261del	p.Phe1088SerfsTer2	p.F1088Sfs*2	COSV99029757	rs267608078	2	2	0	1	1	0.7	19
SMC1A	chrX	53403591	EMM06	NDMM	c.2395C>T	p.Arg799Trp	p.R799W	COSV59129600	NA	3	2	1	0.7	1	0.9	78
TP53	chr17	7676047	EMM06	NDMM	c.322G>A	p.Gly108Ser	p.G108S	COSV52862450	rs587782461	1	1	0	1	1	0.9	39
TP53	chr17	7676077	EMM06	NDMM	c.292C>T	p.Pro98Ser	p.P98S	COSV52787732	rs1597374015	1	1	0	1	1	0.9	33
KRAS	chr12	25245351	EMM07	EMM	c.34G>C	p.Gly12Arg	p.G12R	COSV55497582	rs121913530	2	1	1	0.5	0.9	0.4	180
KRAS	chr12	25245347	EMM07	NDMM	c.38G>A	p.Gly13Asp	p.G13D	COSV55497388	rs112445441	2	1	1	0.5	0.9	0.6	80
ERBB4	chr2	211702093	EMM08	EMM	c.1363G>A	p.Ala455Thr	p.A455T	COSV99038372	rs762866612	2	1	1	0.5	0.9	0	0
KRAS	chr12	25245347	EMM08	EMM	c.38G>A	p.Gly13Asp	p.G13D	COSV55497388	rs112445441	2	1	1	0.5	0.8	0.3	31
STAG2	chrX	124094020	EMM08	EMM	c.3581G>A	p.Arg1194Gln	p.R1194Q	NA	rs756126724	3	2	1	0.7	0.8	0.003	333
TP53	chr17	7673781	EMM08	EMM	c.839G>A	p.Arg280Lys	p.R280K	COSV52666248	rs121912660	1	1	0	1	1	0.9	54
ERBB4	chr2	211702093	EMM08	NDMM	c.1363G>A	p.Ala455Thr	p.A455T	COSV99038372	rs762866612	2	1	1	0.5	1	na	na
KRAS	chr12	25245347	EMM08	NDMM	c.38G>A	p.Gly13Asp	p.G13D	COSV55497388	rs112445441	2	1	1	0.5	1	na	na
STAG2	chrX	124094020	EMM08	NDMM	c.3581G>A	p.Arg1194Gln	p.R1194Q	NA	rs756126724	3	2	1	0.7	0.9	na	na
BRAF	chr7	140753353	EMM09	EMM	c.1902T>A	p.Asp634Glu	p.D634E	COSV56082626	rs121913337	2	1	1	0.5	0.9	0	9
KRAS	chr12	25227334	EMM09	EMM	c.190T>G	p.Tyr64Asp	p.Y64D	COSV55579983	NA	2	1	1	0.5	0.9	0.4	72
ATM	chr11	108326163	EMM10	EMM	c.6913C>T	p.Gln2305Ter	p.Q2305*	NA	rs1282099124	2	1	1	0.5	0.8	1	8
FGFR3	chr4	1801837	EMM10	EMM	c.742C>T	p.Arg248Cys	p.R248C	COSV53390662	rs121913482	3	2	1	0.7	0.7	1	1929
ATM	chr11	108326163	EMM10	NDMM	c.6913C>T	p.Gln2305Ter	p.Q2305*	NA	rs1282099124	2	1	1	0.5	0.9	0.7	25
FGFR3	chr4	1801837	EMM10	NDMM	c.742C>T	p.Arg248Cys	p.R248C	COSV53390662	rs121913482	3	2	1	0.7	0.9	1	2152
KRAS	chr12	25245350	EMM11	EMM	c.35G>T	p.Gly12Val	p.G12V	COSV55497419	rs121913529	3	2	1	0.7	1	0.5	205
FGFR3	chr4	1801726	EMM11	NDMM	c.722A>G	p.Tyr241Cys	p.Y241C	COSV53427887	NA	2	1	1	0.5	0.4	0.4	607
KRAS	chr12	25245350	EMM11	NDMM	c.35G>T	p.Gly12Val	p.G12V	COSV55497419	rs121913529	2	1	1	0.5	0.3	0.2	29
KRAS	chr12	25245351	EMM11	NDMM	c.34G>T	p.Gly12Cys	p.G12C	COSV55497469	rs121913530	2	1	1	0.5	0.2	0	29
ATR	chr3	142550155	EMM12	EMM	c.2953C>A	p.Pro985Thr	p.P985T	COSV63390816	NA	2	1	1	0.5	1	0.7	12
HRAS	chr11	534285	EMM12	EMM	c.38G>T	p.Gly13Val	p.G13V	COSV54237051	rs104894226	3	2	1	0.7	1	0.8	52
NRAS	chr1	114713909	EMM12	EMM	c.181C>A	p.Gln61Lys	p.Q61K	COSV54736310	rs121913254	2	1	1	0.5	1	0.5	285
TP53	chr17	7674872	EMM12	EMM	c.659A>G	p.Tyr220Cys	p.Y220C	COSV52661282	rs121912666	2	2	0	1	1	1	111
KRAS	chr12	25225628	EMM13	EMM	c.436G>C	p.Ala146Pro	p.A146P	COSV55541748	rs121913527	1	1	0	1	1	1	86
TP53	chr17	7673828	EMM13	EMM	c.787_792del	p.Asn263_Leu264del	p.N263_L264del	NA	novel	2	2	0	1	1	1	120
KRAS	chr12	25227342	EMM14	EMM	c.182A>T	p.Gln61Leu	p.Q61L	COSV55504296	rs121913240	3	2	1	0.7	1	0.7	29
KRAS	chr12	25227342	EMM14	NDMM	c.182A>T	p.Gln61Leu	p.Q61L	COSV55504296	rs121913240	3	2	1	0.7	1	0.6	93

Supplementary Table 5: Univariate, multivariate cox model and competing risk model predicting the risk of EMM development using co[illegible]

Baseline characteristics		N (%)	Hazard Ratio		
			CPH univariable	CPH multivariable	competing risks multivariable
**age**	≤ 65	447 (63.9)	-	-	-
	> 65	252 (36.1)	1.49 (0.99-2.25, p=0.058)	1.44 (0.94-2.20, p=0.097)	1.39 (0.90-2.15, p=0.140)
**ISS stage**	1	269 (38.5)	-	-	-
	2	246 (35.2)	0.86 (0.52-1.42, p=0.557)	0.79 (0.48-1.33, p=0.380)	0.78 (0.47-1.29, p=0.330)
	3	184 (26.3)	1.36 (0.83-2.22, p=0.219)	1.14 (0.69-1.90, p=0.610)	1.06 (0.64-1.76, p=0.830)
**LDH level**	Normal	383 (54.8)	-	-	-
	Low	182 (26.0)	0.87 (0.53-1.41, p=0.567)	0.89 (0.54-1.45, p=0.631)	0.91 (0.56-1.47, p=0.690)
	High	134 (19.2)	0.91 (0.51-1.62, p=0.745)	0.94 (0.52-1.69, p=0.837)	0.92 (0.51-1.67, p=0.790)
**1q21 gain/amp + mut KRAS**	absent	314 (44.9)	-	-	-
	1q21 gain/amp	208 (29.8)	1.52 (0.92-2.52, p=0.101)	1.39 (0.83-2.33, p=0.209)	1.37 (0.83-2.24, p=0.220)
	1q21 gain/amp + mut KRAS	53 (7.6)	**2.73 (1.43-5.23, p=0.002)**	**2.41 (1.23-4.73, p=0.011)**	**2.36 (1.22-4.58, p=0.011)**
	mut KRAS	124 (17.7)	1.40 (0.77-2.52, p=0.270)	1.40 (0.77-2.54, p=0.267)	1.41 (0.79-2.53, p=0.250)
**del 13q**	absent	352 (50.4)	-	-	-
	present	347 (49.6)	1.33 (0.88-2.01, p=0.178)	1.22 (0.79-1.87, p=0.374)	1.17 (0.78-1.75, p=0.450)
**del17p**	absent	629 (90.0)	-	-	-
	present	70 (10.0)	0.78 (0.36-1.69, p=0.531)	0.80 (0.36-1.76, p=0.581)	0.80 (0.37-1.72, p=0.570)
